# A Bibliometric Analysis of Research Trends in Paediatric Pelvic-Ureteric Junction Obstruction

**DOI:** 10.7759/cureus.97317

**Published:** 2025-11-20

**Authors:** Alvin Tung Yong Zong, Oon Chong Chew, Wei Kang Yap

**Affiliations:** 1 Urology, Mersey and West Lancashire Teaching Hospitals NHS Trust, Prescot, GBR; 2 Urology, University College London Hospitals NHS Foundation Trust, London, GBR; 3 Paediatrics, Barts Health NHS Trust, London, GBR

**Keywords:** bibliometric analysis, minimally invasive, paediatric, pelvic-ureteric junction obstruction, research trends

## Abstract

Pelvic-ureteric junction obstruction (PUJO) is one of the leading causes of renal impairment in the paediatric population. Over the past few decades, the diagnostic and therapeutic landscape in this field has evolved significantly. We conducted a comprehensive quantitative bibliometric analysis of research trends in PUJO over the last four decades. A total of 2019 articles published between 1985 and 2025 were retrieved from Web of Science Core Collection and analysed using the Bibliometrix R package (version 4.3.2; K-Synth Srl, Naples, Italy) and VOSviewer (Centre for Science and Technology Studies, Leiden University, The Netherlands). The annual number of publications has increased steadily over the study period. The United States was the most productive country (n=574), while the *Journal of Pediatric Urology* was the most active journal (n=255). Peters, CA, was identified as the most influential author with 1373 citations and 49.00 average citations per publication. Keyword analysis has revealed a shift away from traditional approaches like open surgery and ultrasonography to modern surgical and imaging techniques such as robotic surgery and magnetic resonance urography. Future research is trending towards refining minimally invasive approaches, understanding their long-term outcomes, and exploring novel biomarkers.

## Introduction and background

Pelvic ureteric junction obstruction (PUJO), also referred to as ureteropelvic junction obstruction, is characterised by impaired urine flow from the renal pelvis to the proximal ureter [1]. It represents the most frequent pathological cause of antenatal hydronephrosis [2], occurring in approximately one in 750-1500 live births, with a higher incidence in males [3] and left-sided predominance in nearly two-thirds of cases [4]. Urinary tract obstruction remains one of the leading causes of renal impairment in the paediatric population, and, if untreated, PUJO may result in recurrent urinary tract infections, progressive renal dysfunction, and pain [5]. The pathogenesis of PUJO is multifactorial, encompassing embryological, anatomical, functional, and histopathological abnormalities [6]. Increasing evidence suggests that molecular and genetic factors may contribute to abnormal junctional development and defective peristaltic mechanisms [7].

The majority of cases are now detected antenatally through routine ultrasound scanning between 18 and 20 weeks of gestation [8]. Although antenatal detection has facilitated early evaluation, differentiation between obstructive and non-obstructive dilatation remains challenging. Traditional diagnostic modalities, such as ultrasonography and diuretic renography, have limited accuracy in predicting renal deterioration [9], whereas magnetic resonance urography offers superior anatomical detail and functional assessment, albeit with higher cost and the need for sedation [10]. Recent advances in urinary biomarkers hold promise for non-invasive assessment of renal injury and may guide clinical decision-making [11].

Since the introduction of the dismembered pyeloplasty by Anderson and Hynes in 1949 [12], management of PUJO has evolved from routine surgical correction to a more selective, function-based approach. Nevertheless, the optimal timing of intervention and long-term outcomes remains subject to ongoing debate. The advent of robotics has meant that the approach to pyeloplasty has evolved from the early open-based approach to more minimally invasive techniques [13,14].

Despite the significant evolution in the diagnosis and management of paediatric PUJO, no formal assessment of the current research landscape in this field has been established. In this study, we employ a quantitative bibliometric analysis methodology to comprehensively evaluate four decades of global research trends in paediatric PUJO. We aim to identify influential publications and contributors, explore the evolution of research trends, and highlight emerging themes and gaps to inform future research directions. 

## Review

Methods

Data Source and Search Strategy

A search of the Web of Science Core Collection (WoSCC) database was performed on August 21, 2025. While other databases like Scopus and PubMed exist, WoSCC is frequently used for high-impact bibliometric analyses as it provides comprehensive and standardized citation data, which is essential for network and influence analysis. Hence, WoSCC was selected as the sole database for this analysis. Its stringent journal selection criteria also ensure a comprehensive dataset of high-quality literature. The following search query was used: TS=(("ureteropelvic junction obstruction" OR "pelvic ureteric junction obstruction" OR "upj obstruction" OR "pujo" OR "pyeloplasty") AND (pediatric OR paediatric OR child OR children OR infant* OR neonat* OR adolescen*)).

Inclusion and Exclusion Criteria

**Figure 1:**
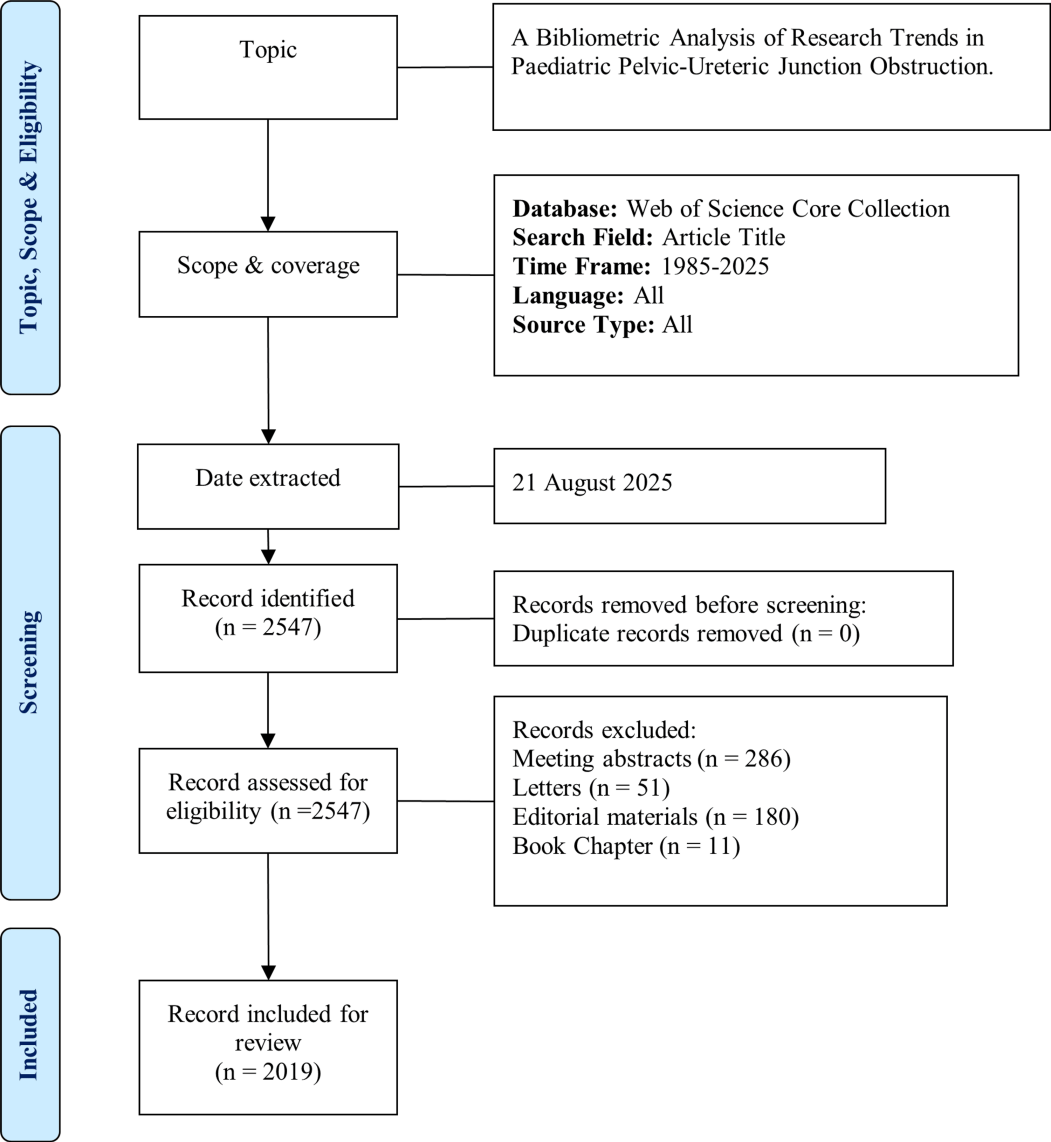
Flow diagram of the search strategy.

Articles published between 1985 and 2025 were selected for the analysis. This extended timeframe, encompassing four decades of research on this topic, ensures the inclusion of foundational studies on paediatric PUJO and allows for an analysis of the temporal evolution of its research directions and themes. Inclusion criteria include original articles, case reports, and review articles. Meeting abstracts, letters, editorial materials, and book chapters were excluded from the study. No language restrictions were applied. The remaining articles were then independently screened by two authors (ATYZ and OCC) for relevance to the subject. Figure 1 summarises our search strategy with a flowchart.

Data Analysis and Visualization

Initial data extraction yielded 2547 records. After screening, 2019 articles were exported and included in the final review. Performance analysis was performed using the Bibliometrix package (version 4.3.2; K-Synth Srl, Naples, Italy) based on the R language (version 4.4.2; R Foundation for Statistical Computing, Vienna, Austria, https://www.R-project.org/) and accessed through RStudio software. Visual maps of authors, countries, and networks were generated using VOSviewer version 1.6.20 (Centre for Science and Technology Studies, Leiden University, The Netherlands).

Results 

Overview of Publications

**Figure 2:**
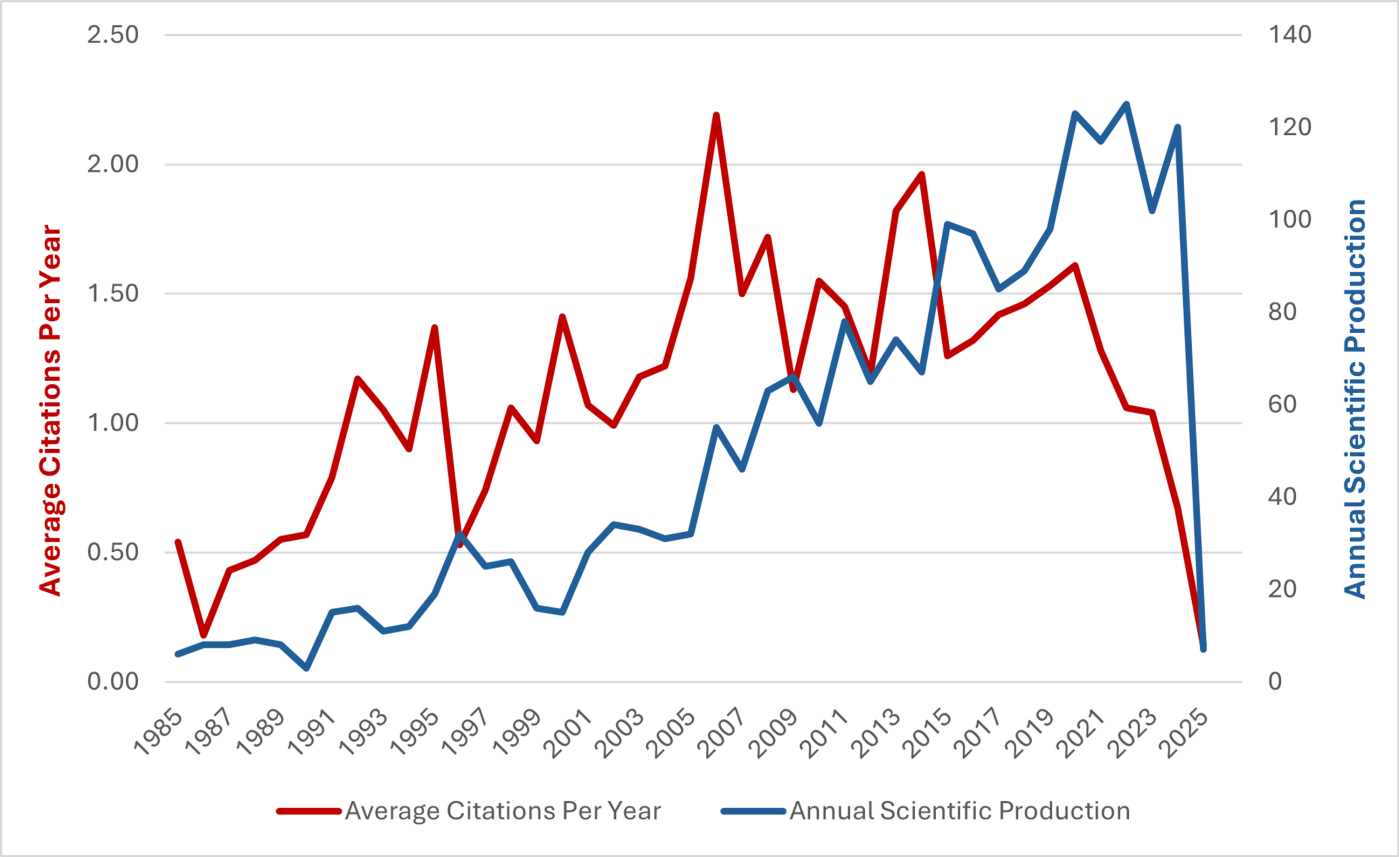
Annual scientific production and average article citation per year in paediatric pelvic-ureteric junction obstruction research from 1985 to 2025.

A total of 2019 publications were included in the final review, comprising 1794 original research articles and 225 review articles. The dataset included the work of 7,798 authors from 1,886 institutions across 71 countries, with publications appearing in 375 distinct journals. There were 2493 keywords across all articles. In terms of annual scientific production, there is a steady increase in the number of publications from 1985 to 2024, with a peak of 125 articles published in 2022. The average number of citations per year has also seen an increase since 1985, with a peak of 2.19 average citations in 2006, followed by another peak of 1.96 average citations in 2014. Figure 2 summarises the publication trend in paediatric PUJO from 1985 to 2025.

Most Active Journals

The most active journal in the field of paediatric PUJO research is the *Journal of Pediatric Urology*, with 255 (12.6%) publications and an impact factor of 1.9 (Table 1). The *Journal of Urology*, published by the American Urological Association, is the second most active journal with 190 (9.4%) publications, followed by *Urology* with 126 (6.2%) publications. Other notable journals in the top 10 include *Journal of Endourology*, *Frontiers in Pediatrics*, and *Pediatric Surgery International*.

**Table 1:** Top 10 Most Active Journals in Paediatric Pelvic-Ureteric Junction Obstruction Research (1985-2025).

Rank	Journal Title	Publications, n (%)	Impact Factor
1	Journal of Pediatric Urology	255 (12.6%)	1.9
2	Journal of Urology	190 (9.4%)	7.6
3	Urology	126 (6.2%)	2.0
4	Journal of Endourology	64 (3.2%)	2.8
5	Frontiers in Pediatrics	60 (2.9%)	2.0
6	Pediatric Surgery International	59 (2.9%)	1.6
7	Journal of Laparoendoscopic & Advanced Surgical Techniques	50 (2.5%)	1.1
8	Pediatric Nephrology	43 (2.1%)	2.6
9	BJU international	42 (2.1%)	4.4
10	Journal of Pediatric Surgery	39 (1.9%)	2.5

Most Active Institutions

Table 2 illustrates the top 10 institutions in paediatric PUJO research. The All India Institute of Medical Science (AIIMS) leads the field with 85 (4.2%) publications, highlighting the institution’s emphasis on paediatric urological disorders. This is followed by Canadian institutions, including the University of Toronto (n=72, 3.6%) and The Hospital for Sick Children (n=70, 3.5%). Leading United States institutions such as the University of Chicago (n=61, 3.0%) and Harvard University (n=58, 2.9%) rank among the top five institutions in this field, underscoring North America’s sustained leadership in paediatric urological research.

**Table 2:** Top 10 Most Active Institutions in Paediatric Pelvic-Ureteric Junction Obstruction Research (1985-2025).

Rank	Institution	Country	Publications, n (%)
1	All India Institute of Medical Science	India	85 (4.2%)
2	University of Toronto	Canada	72 (3.6%)
3	The Hospital for Sick Children	Canada	70 (3.5%)
4	University of Chicago	United States	61 (3.0%)
5	Harvard University	United States	58 (2.9%)
6	McMaster University	Canada	56 (2.8%)
7	University of Virginia	United States	55 (2.7%)
8	Children’s Hospital of Philadelphia	United States	54 (2.7%)
9	Istanbul University	Turkey	50 (2.5%)
10	University of Pennsylvania	United States	49 (2.4%)

**Figure 3:**
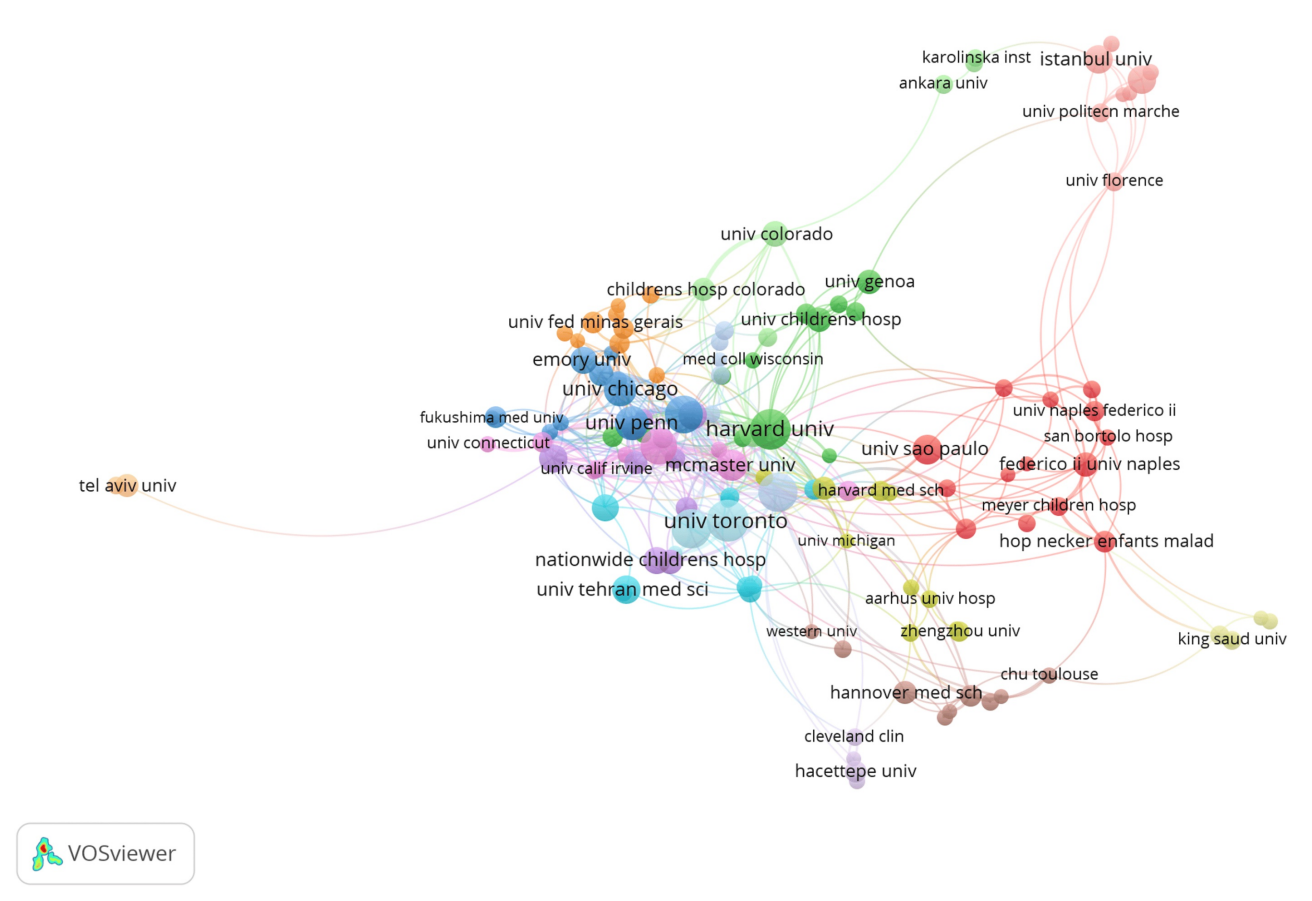
Institutional Collaboration Network. Image generated using VOSviewer version 1.6.20 (Centre for Science and Technology Studies, Leiden University, The Netherlands)

The institutional collaboration network (Figure 3) reveals several distinct clusters of institutional co-authorship. The dense red cluster on the right suggests a strong Western European network, led by prominent institutions such as the University of Naples Federico II and the University of Florence in Italy, and the Necker-Enfants Malades Hospital in France. A central cluster formed by blue, green, and purple clusters is comprised mainly of North American institutions such as the University of Pennsylvania, the University of Chicago, and the University of Toronto. The green cluster of institutions, led by Harvard University, appears to play a significant role in bridging research collaboration between European and North American institutions.

Most Productive and Influential Countries

As seen in Table 3, the top three most productive countries in this field of research are the United States (574, 28.4%), China (174, 8.6%), and India (141, 7.0%), respectively. The top three countries with the highest total citations are the United States (14,675), followed by China (1,885) and the United Kingdom (1,729). In terms of average citations, however, Sri Lanka holds the highest number of average citations with 44.5 citations per publication.

**Table 3:** Top 10 Most Active Countries in Paediatric Pelvic-Ureteric Junction Obstruction Research (1985-2025).

Rank	Country	Publications, n (%)	Total citations	Average citations
1	United States	574 (28.4%)	14675	25.60
2	China	174 (8.6%)	1885	10.80
3	India	141 (7.0%)	1220	8.70
4	Turkey	106 (5.3%)	1214	11.50
5	Italy	101 (5.0%)	1501	14.90
6	Germany	93 (4.6%)	1117	12.00
7	France	83 (4.1%)	1589	19.10
8	United Kingdom	79 (3.9%)	1729	21.90
9	Canada	75 (3.7%)	1294	17.30
10	Iran	57 (2.3%)	341	7.30

Most Productive and Influential Authors

Peters, C.A., with 28 (1.39%) publications and 1,373 total citations, is the leading contributor to paediatric PUJO research and is particularly recognized for pioneering minimally invasive surgical approaches and protocol development that have shaped modern paediatric urological practice. He is followed by Lorenzo, AJ, with 26 (1.29%) articles, and Esposito, C, with 25 (1.24%) articles. Gundeti, MS (n = 24, 1.19%), and Casale, P (n = 22, 1.09%) complete the top five most productive authors (Table 4). In terms of influence, Nguyen, HT, and Peters, CA, have the highest number of average citations, with 53.63 and 49.00 citations per publication, respectively.

**Table 4:** Top 10 Most Active Authors in Paediatric Pelvic-Ureteric Junction Obstruction Research (1985-2025).

Rank	Author	Publications, n (%)	Total Citations	Average Citations
1	Peters, CA	28 (1.39%)	1373	49.00
2	Lorenzo, AJ	26 (1.29%)	432	16.62
3	Esposito, C	25 (1.24%)	330	13.20
4	Gundeti, MS	24 (1.19%)	385	16.04
5	Casale, P	22 (1.09%)	584	26.55
6	Noh, PH	20 (0.99%)	368	18.40
7	Escolino, M	19 (0.94%)	258	13.58
8	Blanc, T	18 (0.89%)	244	13.55
9	Masieri, L	17 (0.84%)	233	13.70
10	Nguyen, HT	16 (0.79%)	858	53.63

**Figure 4:**
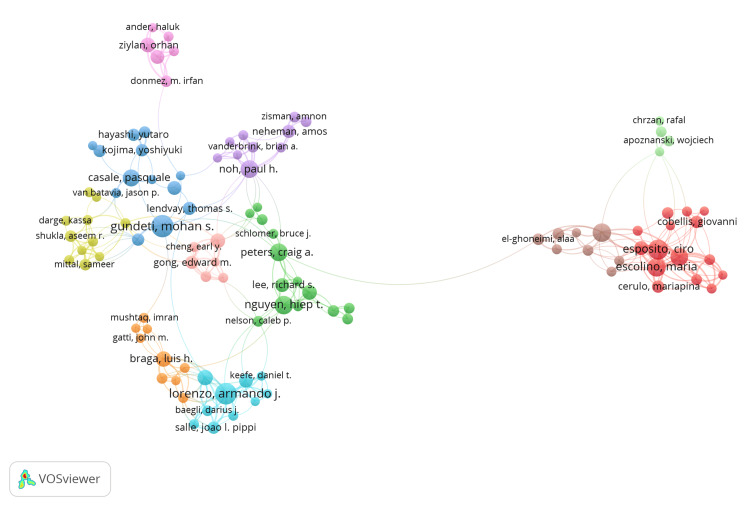
Author Collaboration Network. Image generated using VOSviewer version 1.6.20 (Centre for Science and Technology Studies, Leiden University, The Netherlands)

Figure 4 highlights the author collaboration network in this field of research, with the size of nodes representing author productivity. Only authors with a minimum of five published documents were included, and a full counting method was used. Several large clusters can be seen in the network. A separate large red cluster on the right, led by Esposito, C, highlights the extensive collaboration between European authors, although their collaboration network appears to be more geographically isolated. On the left side of the figure, network analysis reveals close collaboration between prominent North American authors such as Gundeti, M, Peters, CA, and Lorenzo, AJ.

Analysis of Highly Cited Documents

Table 5 illustrates the top 10 most influential publications in paediatric PUJO research. The most highly cited article in this field is “The Society for Fetal Urology Consensus Statement on the Evaluation and Management of Antenatal Hydronephrosis”, published in 2010 by Nguyen et al. [15], with 457 total citations and 28.56 total citations per year. This is followed by “Transumbilical Single-port Surgery: Evolution and Current Status” by Canes et al. [16] with 327 citations and “Antenatal Hydronephrosis as a Predictor of Postnatal Outcome: A Meta-analysis” by Lee et al. [17] with 316 citations.

**Table 5:** Top 10 Most Influential Publications in Paediatric Pelvic-Ureteric Junction Obstruction Research (1985-2025) TC: total citations

Rank	First Author	Title	Year	TC	TC/ Year
1	Nguyen, HT	The Society for Fetal Urology Consensus Statement on the Evaluation and Management of Antenatal Hydronephrosis [15]	2010	457	28.56
2	Canes, D	Transumbilical Single-port Surgery: Evolution and Current Status [16]	2008	327	18.17
3	Lee, RS	Antenatal Hydronephrosis as a Predictor of Postnatal Outcome: A Meta-analysis [17]	2006	316	15.80
4	Lee, RS	Pediatric Robot-Assisted Laparoscopic Dismembered Pyeloplasty: Comparison with a Cohort of Open Surgery [18]	2006	251	12.55
5	Decramer, S	Predicting the Clinical Outcome of Congenital Unilateral Ureteropelvic Junction Obstruction in Newborn by Urinary Proteome Analysis [19]	2006	206	10.30
6	Peters, CA	Pediatric Laparoscopic Dismembered Pyeloplasty [20]	1995	204	6.58
7	Xu, N	Comparison of Retrograde Balloon Dilatation and Laparoscopic Pyeloplasty for Treatment of Ureteropelvic Junction Obstruction: Results of a 2-Year Follow-Up [21]	2016	180	18.00
8	Autorino, R	Robot-assisted and Laparoscopic Repair of Ureteropelvic Junction Obstruction: A Systematic Review and Meta-analysis [22]	2014	177	14.75
9	Chertin, B	Conservative Treatment of Ureteropelvic Junction Obstruction in Children with Antenatal Diagnosis of Hydronephrosis: Lessons Learned after 16 Years of Follow-Up [23]	2006	162	8.10
10	Meretyk, I	Endopyelotomy: Comparison of Ureteroscopic Retrograde and Antegrade Percutaneous Techniques [24]	1992	160	4.71

Keyword Analysis

**Figure 5:**
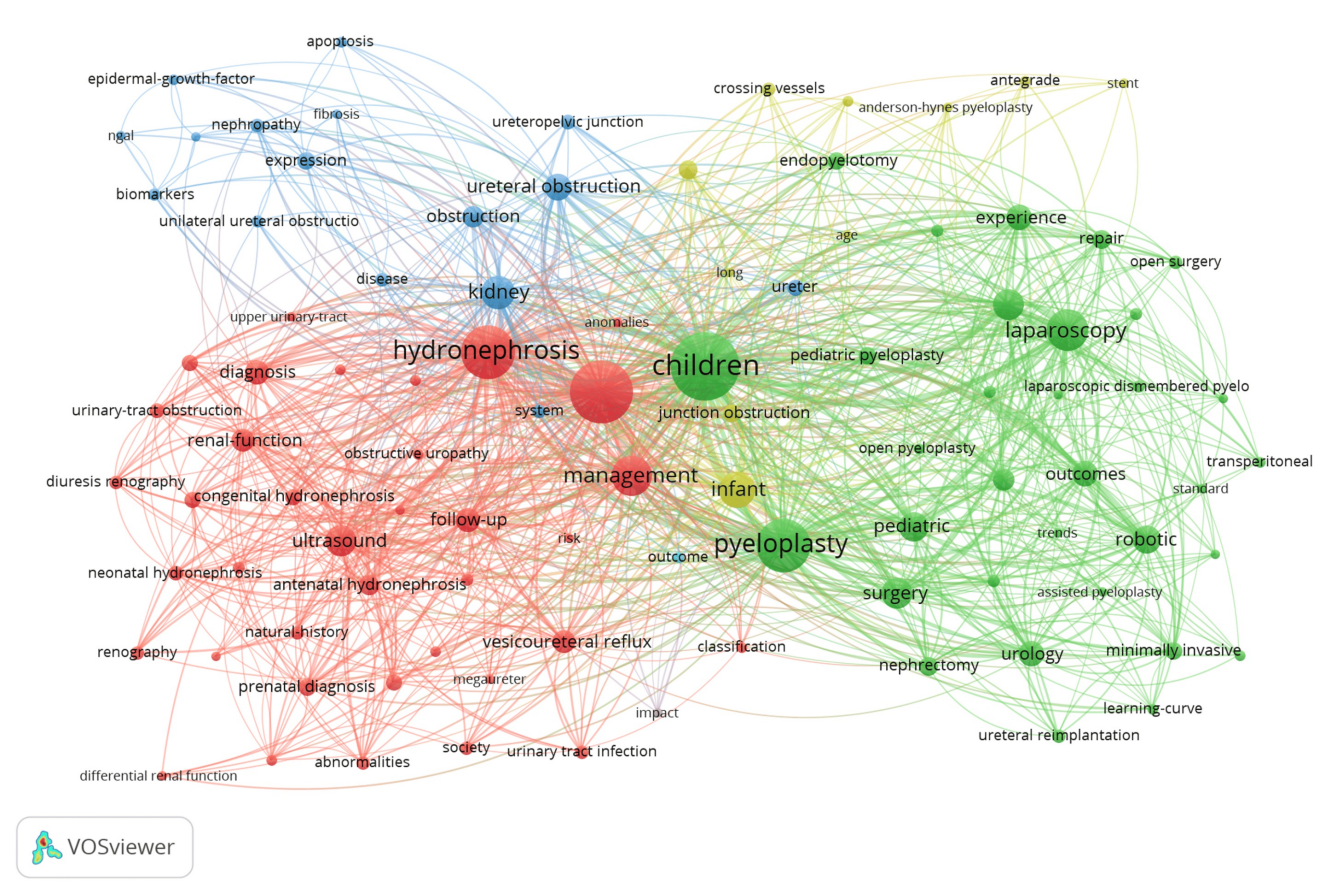
Keyword Co-occurrence Network. Image generated using VOSviewer version 1.6.20 (Centre for Science and Technology Studies, Leiden University, The Netherlands)

Our analysis revealed a total of 3777 keywords, with 120 keywords appearing 20 times or more. Figure 5 illustrates the keyword co-occurrence network in the field of paediatric PUJO research, with a minimum threshold of 20 co-occurrences set for keywords. Four main clusters can be seen: red, blue, green, and yellow. The red cluster is the largest in the network and focuses on the diagnosis of PUJO in the paediatric population, with keywords such as “antenatal hydronephrosis”, “ultrasound”, and “renography”. The next major cluster is the green cluster, and this represents key treatment options for PUJO, including “open pyeloplasty”, “nephrectomy”, and “endopyelotomy”. A third, distinct blue cluster delves into the pathophysiology and complications of PUJO, with keywords such as “epidermal-growth factor”, “ureteral obstruction”, and “fibrosis”.

**Figure 6:**
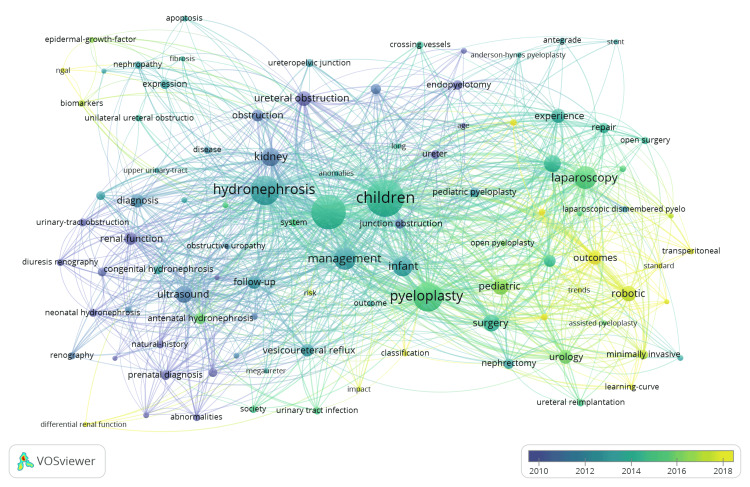
Keyword Overlay Analysis. Image generated using VOSviewer version 1.6.20 (Centre for Science and Technology Studies, Leiden University, The Netherlands)

The overlay analysis in Figure 6 demonstrates the temporal evolution of research themes in this field. Older keywords are represented by blue nodes (2010-2012) and focus on fundamental investigations such as “ultrasound” and “diuresis renography”, as well as early surgical techniques such as “open surgery”. More recently (yellow nodes), the research trends have shifted towards newer surgical techniques, such as "robotic" surgery, "minimally invasive" techniques, and the associated "learning curve" and "trends".

Discussion

This is the first study, to our knowledge, to provide a comprehensive overview of the global research landscape in paediatric PUJO spanning four decades. There is a growing interest in this field, with the annual publication volume showing consistent growth from eight papers in 1985 to 125 in 2022. This is an optimistic sign as PUJO remains one of the leading causes of renal impairment in the paediatric population, and extensive research in this field can help ensure optimal outcomes for these patients.

Research in this field is dominated by contributions from North American institutions, accounting for 32.1% of the total publication volume. Institutions from the United States and Canada make up eight of the top 10 most active institutions. This phenomenon can be attributed to institutions such as the University of Toronto and Harvard University, which are high-volume tertiary paediatric urology centres with a significant research culture. Furthermore, as established in our findings, North American institutions are early adopters of newer minimally invasive and robotic surgical approaches to managing PUJO, driving much higher publication output.

Encouragingly, there is an increasing emergence of China and India as major contributors in this field, highlighting the globalisation of research. There is, however, geographical isolation in terms of research output, with institutions from China and India not part of the major collaboration network seen in Figure 3. This is potentially due to linguistic differences; however, it could also be a reflection that American and European institutions are more likely to have established collaboration networks, research conferences, and share similar funding bodies. Furthermore, different healthcare systems and patient data storage between different countries may complicate international data sharing.

The most prominent authors in this field are Peters, CA, Lorenzo, AJ, and Gundeti, MS, as evidenced by their publication volume and central location in our author collaboration network analysis (Figure 4). Peters, CA, from The University of Texas Southwestern Medical Center (UT Southwestern), is the most influential author, with 1373 citations from 28 publications, and an average of 49.00 citations per publication. Similarly, there is significant geographical isolation between authors, with two large but separate clusters of North American and European authors seen in the author collaboration network. We believe that greater intercontinental collaboration should be fostered to further accelerate research in this field and to ensure novel findings are applicable to the global population.

Temporal Evolution of PUJO Research

The keyword analysis (Figures 5, 6) has clearly highlighted the key research themes and the temporal evolution of research in this field. The main themes, as demonstrated by the coloured clusters, can be broadly subdivided into diagnostic investigations, surgical techniques, and pathophysiology of the disease. Temporal analysis indicates that research activity in the early 2000s concentrated on established diagnostic tools such as antenatal ultrasonography and diuretic renography, alongside conventional surgical interventions such as open pyeloplasty and endopyelotomy. Subsequent citation trends suggest a progressive transition toward minimally invasive and robotic methodologies during 2015-2025. The Anderson-Hynes dismembered pyeloplasty technique, first described in 1949 [12], remains the gold-standard surgical technique for treating PUJO. With the introduction of robotic surgery, there has been a clear shift in research focus, with more recent articles (2020-2025) using keywords such as “robotics”, “minimally invasive”, and “learning curve”, highlighting a shift in surgical techniques towards robotic and laparoscopic-based methods. There is also a shift in diagnostic investigations for PUJO, with keywords such as "magnetic resonance pyrography" and biomarkers such as “epidermal growth factor” and “NGAL” (neutrophil gelatinase-associated lipocalin) now trending.

Clinical and Research Implications

Our findings have direct implications for both clinicians and researchers. The clear temporal shift from "open surgery" to "robotic" and "minimally invasive" techniques, as seen in our overlay analysis (Figure 6), signifies that these approaches are moving from niche to standard of care. There is now clear evidence that minimally invasive surgical techniques minimise surgical morbidity, reduce hospital stays, and enhance recovery in patients undergoing these procedures [25,26]. For clinical practice, this highlights the critical need for structured training programs for the next generation of surgeons to navigate the associated learning curve of minimally invasive and robotic surgery. Interestingly, the preferred gold-standard surgical technique at present remains the Anderson-Hynes dismembered pyeloplasty [27], despite being first described almost 70 years ago [12], indicating that innovation has focused on technological adaptation rather than procedural technique. 

From a research perspective, our analysis identifies several clear future priorities. The emergence of keywords like "biomarkers", "epidermal growth factor", and "NGAL" points to a growing research interest towards molecular diagnostics and precision medicine in PUJO. NGAL is a novel urinary biomarker, and there is currently growing interest in its potential as a marker for ureteric obstruction [28]. Additionally, magnetic resonance urography has increasingly been shown to offer superior anatomical detail and functional assessment compared to traditional methods [10,29]. Future studies must now move to validate whether these novel biomarkers or advanced imaging methods can reliably predict the need for intervention and replace or augment traditional diagnostic methodology. This is particularly important in the paediatric population, where less invasive tests are preferred.

## Conclusions

This study presents a concise bibliometric analysis of research trends in paediatric pelvic-ureteric junction obstruction from 1985-2025. Our analysis shows an increasing research effort by the urology community all over the world in this field, as evidenced by the increasing number of publications. However, there remains significant geographical isolation between research groups. The evolution of robotic surgery has shifted research focus in the surgical treatment of PUJO to involve robotic or laparoscopic surgery instead of open surgery. Additionally, the increase in research within this field has highlighted new diagnostic investigations that could potentially benefit diagnostic and medical management going forward.
